# PIP_3_-binding proteins promote age-dependent protein aggregation and limit survival in *C. elegans*

**DOI:** 10.18632/oncotarget.10549

**Published:** 2016-07-12

**Authors:** Srinivas Ayyadevara, Meenakshisundaram Balasubramaniam, Jay Johnson, Ramani Alla, Samuel G. Mackintosh, Robert J. Shmookler Reis

**Affiliations:** ^1^ McClellan Veterans Medical Center, Central Arkansas Veterans Healthcare Service, Little Rock, AR, USA; ^2^ BioInformatics Program, University of Arkansas for Medical Sciences and University of Arkansas at Little Rock, Little Rock, AR, USA; ^3^ Reynolds Institute on Aging/Department of Geriatrics, University of Arkansas for Medical Sciences, Little Rock, AR, USA; ^4^ Department of Biochemistry & Molecular Biology, University of Arkansas for Medical Sciences, Little Rock, AR, USA

**Keywords:** phosphatidylinositol 3-kinase, phosphatidylinositol 3,4,5-triphosphate (PIP_3_), longevity, oxidative stress resistance, protein aggregation

## Abstract

Class-I phosphatidylinositol 3-kinase (PI3K_I_) converts phosphatidylinositol 4,5-bisphosphate (PIP_2_) to phosphatidylinositol 3,4,5-triphosphate (PIP_3_). PIP_3_ comprises two fatty-acid chains that embed in lipid-bilayer membranes, joined by glycerol to inositol triphosphate. Proteins with domains that specifically bind that head-group (e.g. pleckstrin-homology [PH] domains) are thus tethered to the inner plasma-membrane surface where they have an enhanced likelihood of interaction with other PIP_3_-bound proteins, in particular other components of their signaling pathways. Null alleles of the *C. elegans age-1* gene, encoding the catalytic subunit of PI3K_I_, lack any detectable class-I PI3K activity and so cannot form PIP_3_. These mutant worms survive almost 10-fold longer than the longest-lived normal control, and are highly resistant to a variety of stresses including oxidative and electrophilic challenges. Traits associated with *age-1* mutation are widely believed to be mediated through AKT-1, which requires PIP_3_ for both tethering and activation. Active AKT complex phosphorylates and thereby inactivates the DAF-16/FOXO transcription factor. However, extensive evidence indicates that pleiotropic effects of *age-1*-null mutations, including extreme longevity, cannot be explained by insulin like-receptor/AKT/FOXO signaling alone, suggesting involvement of other PIP_3_-binding proteins. We used ligand-affinity capture to identify membrane-bound proteins downstream of PI3K_I_ that preferentially bind PIP_3_. Computer modeling supports a subset of candidate proteins predicted to directly bind PIP_3_ in preference to PIP_2_, and functional testing by RNAi knockdown confirmed candidates that partially mediate the stress-survival, aggregation-reducing and longevity benefits of PI3K_I_ disruption. PIP_3_-specific candidate sets are highly enriched for proteins previously reported to affect translation, stress responses, lifespan, proteostasis, and lipid transport.

## INTRODUCTION

Class-I phosphatidylinositol 3-kinase (PI3K_I_) is the enzyme responsible for converting phosphatidylinositol 4,5-bisphosphate (PIP_2_) to phosphatidylinositol 3,4,5-triphosphate (PIP_3_). In the nematode *C. elegans*, mutations inactivating AGE-1, the catalytic subunit of PI3K_I_, result in increased resistance to a variety of stresses and extension of lifespan [1, 2] by as much as 10-fold [3]. Although PI3K lies in the insulin-like receptor/IRS/PI3K/AKT/FOXO pathway, pleiotropic effects of *age-1*-null mutations greatly exceed those of other disruptions to that signaling cascade, suggesting the possible involvement of other PIP_3_-binding proteins [2, 3]. There are >200 human proteins with Pleckstrin Homology (PH) domains, augmented by additional phosphoinositide-binding-domain families (see below). However, very few proteins in these families have been shown to preferentially bind PIP_3_, and protein homology has proven insufficient to identify PIP_3_-specific binding [4]. In contrast, structural analysis has shown promise for predicting PH-domain binding preferences [5]. We used PIP_3_- and PIP_2_-affinity enrichment to compare proteins from isogenic strains that differ genetically in their ability to make PIP_3_, coupled to proteomic identification of the binding proteins. Molecular modeling of proteins that show preferential binding to PIP_3_ over PIP_2_ allows us to extend structural prediction beyond PH-domain proteins, with the potential for confirmation by functional testing.

Phosphorylated derivatives of phosphatidylinositol (phosphoinositides or PtdInsPs) are key components of multiple signal-transduction complexes that convey external signals to the interior of cells [6-8]. PtdInsPs play critical roles in diverse cellular processes including growth, development, reproduction, cancer and longevity [9-11]. Phosphoinositides comprise 10-20% of phospholipids, but only ~1% of membrane lipids [12]. Proliferating cancer cells have elevated PIP_3_ levels due to elevated activity of PI3K_I_ [13] or reduced activity of the opposing phosphatase, PTEN [14, 15], best known as an anti-oncogene.

Proteins with reported specificity for PIP_3_ include the serine/threonine kinases AKT and PDK-1 [4]; Phospholipase C (PLC) isoforms [16]; atypical protein kinase C, aPKC (e.g., Mζ and ι / λ isoforms) [17]; cytohesins [4]; general receptor for phosphoinositides 1, GRP1 [18]; kindlin3 [19]; IL-2 inducible T-cell kinase, ITK [20]; Skap-hom [21]; and Arf GTPase-activating protein 1 [4, 22]. Protein domains reported to confer PIP_3_-specific binding *in some proteins* (although based on quite variable evidence) include pleckstrin homology (PH) domains [4], Phox-homology (PX) and FYVE domains [23, 24], P2X domains [25], and the SYLF domain [26].

While disruption of insulin and insulin-like signaling (IIS) pathways has been shown to extend lifespan in diverse species, the elimination of class-I PI3K confers at least 4-fold greater life extension than any other IIS mutation [3]. The basis for this heightened dependence on PI3K_I_ remains unresolved, but may be related to the ability of PI3K_I_ mutation to reduce protein aggregation [27], which accompanies normal aging but is elevated and neurotoxic in most or all neurodegenerative diseases. IIS, and in particular PI3K_I_, modulate neuronal processes including learning and neuron survival [28, 29]. In the current study we identified the PIP_3_-binding proteins from *C. elegans* and showed that some are involved in age-related traits including oxidative stress resistance and longevity in normal worms, as well as protein aggregation and associated functional impairment in *C. elegans* models of neurodegeneration-associated proteinopathy.

## RESULTS

### PI3K_I_ contributes to protein aggregation in diverse nematode models

**Figure 1:**
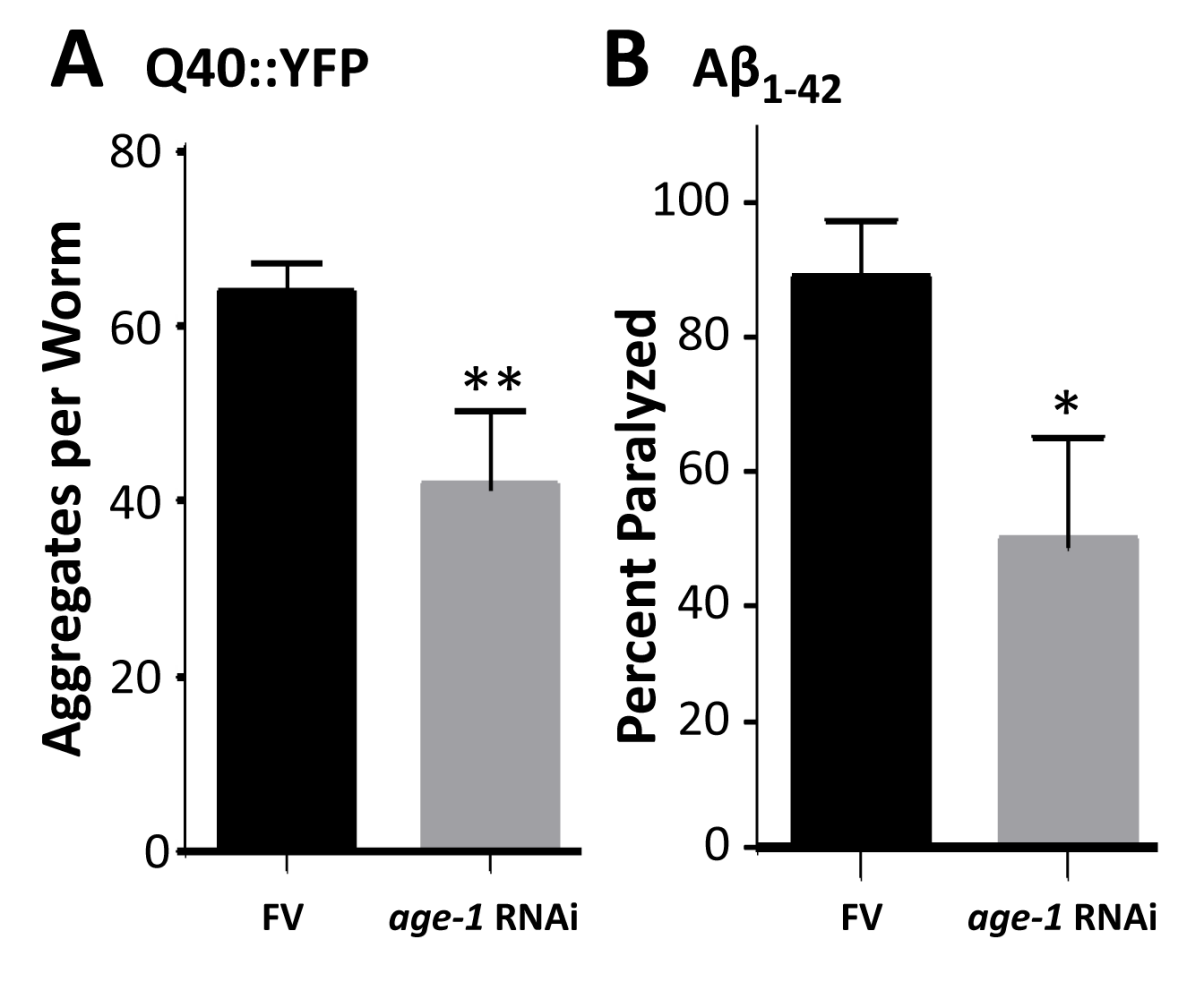
Knock-down of *age-1* reduces Q40::YFP aggregates and protects against paralysis in worms expressing Aβ_**1-42**_ in body-wall muscle **A**. assays of aggregate count in strain AM141 at adult day 4. **B**. assays of paralysis in strain CL4176 48 hr after induction of Aβ_1-42_. *P < 0.02; **P < 0.0001.

Components of the IIS pathway, including PI3K_I_, have been implicated in diverse neuropathologies including Alzheimer's disease [30-32]. PI3K_I_ is a key mediator of protein aggregation [27] and the unfolded protein response [33], leading us to test whether its knockdown (by RNAi targeting the *age-1* gene that encodes the PI3K_I_ catalytic subunit) would rescue nematode** models of protein aggregation. In adult *C. elegans* with muscle expression of a Q40::YFP transgene, *age-1* knockdown reduced the number of fluorescent aggregates by >35% (Figure 1A; *P* < 10 ^−4^). Moreover, in worms expressing human Aβ_1-42_ in muscle, amyloid-induced paralysis declined 46% after *age-1* knockdown (Figure 1B; *P* = 0.02).

### PIP_3_ deficiency reduces the yield of membrane proteins and especially of membrane-associated PIP_3_-binding proteins

**Figure 2:**
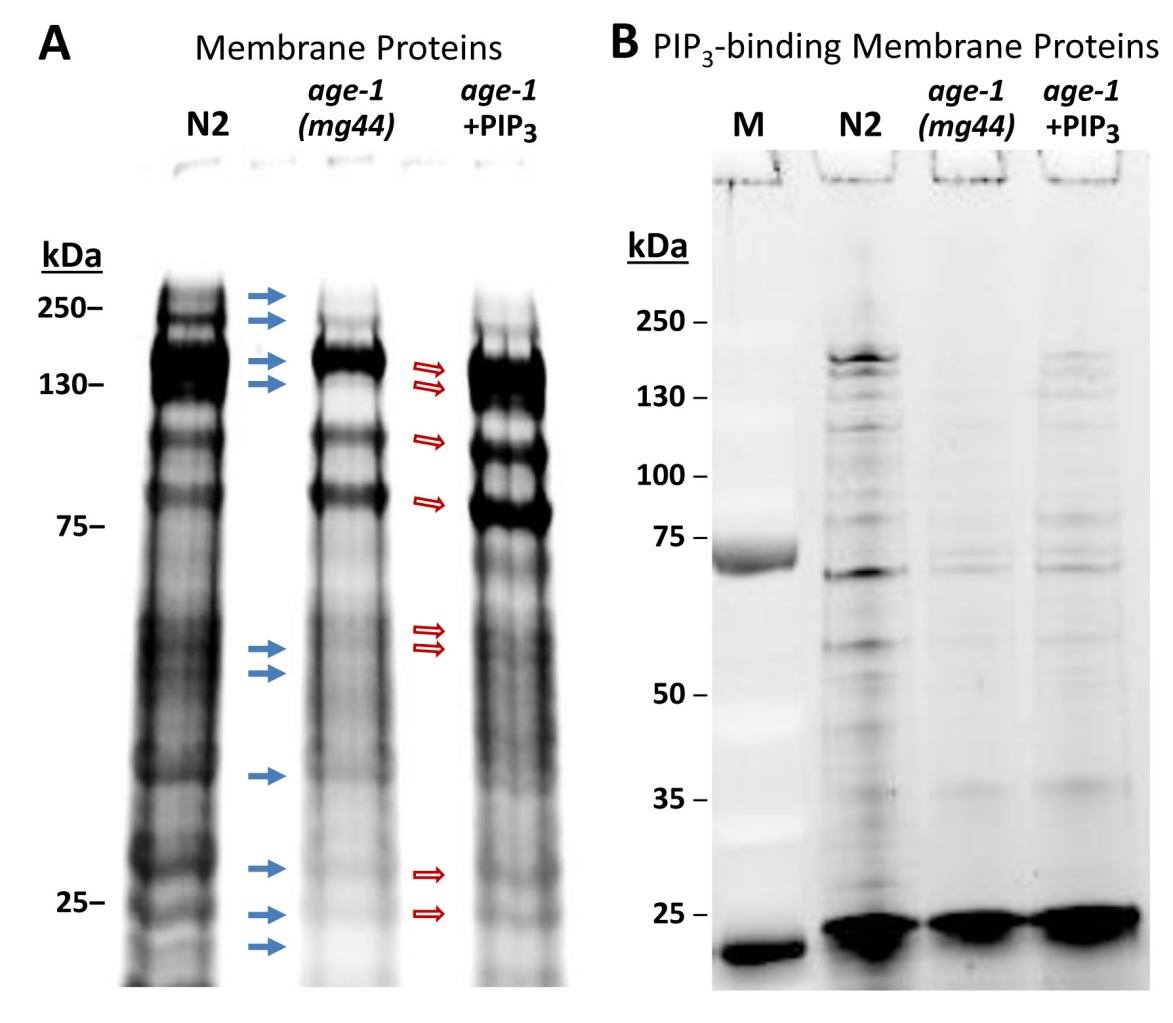
A strong nonsense mutation in the *age-1* gene (allele *mg44)* reduces the recovery of many membrane proteins relative to wild-type controls, whereas feeding PIP_**3**_ to worms restores some bands Polyacrylamide/SDS gels, stained with SYPRO Ruby after electrophoresis, show **A.** isolated membrane proteins, and **B.** proteins recovered from PIP_3_-coated beads, bound after isolation of membrane proteins as in A.

The two fatty-acid chains of PIP_3_ are embedded in the inner plasma membrane, whereas PIP_3_-binding domains such as PH bind the phosphorylated inositol ring that projects into the cytoplasm. In this way, key signaling proteins such as AKT, PDK-1, PLCs, and aPKC are tethered to the inner membrane surface, where they are in proximity to other signaling kinases via clustering [34]. We used PIP_3_-coated agarose beads to isolate PIP_3_-binding proteins from *C. elegans* membranes, in which they are expected to be enriched. Based on staining of electrophoresed proteins, most but not all membrane proteins isolated from wild-type (N2) adults were less abundant in *age-1*-null mutant adults (Figure 2, solid arrows). Many protein bands, not necessarily the same ones, increased in abundance in worms fed a diet supplemented with exogenous PIP_3_ (open arrows, Figure 2).

**Figure 3:**
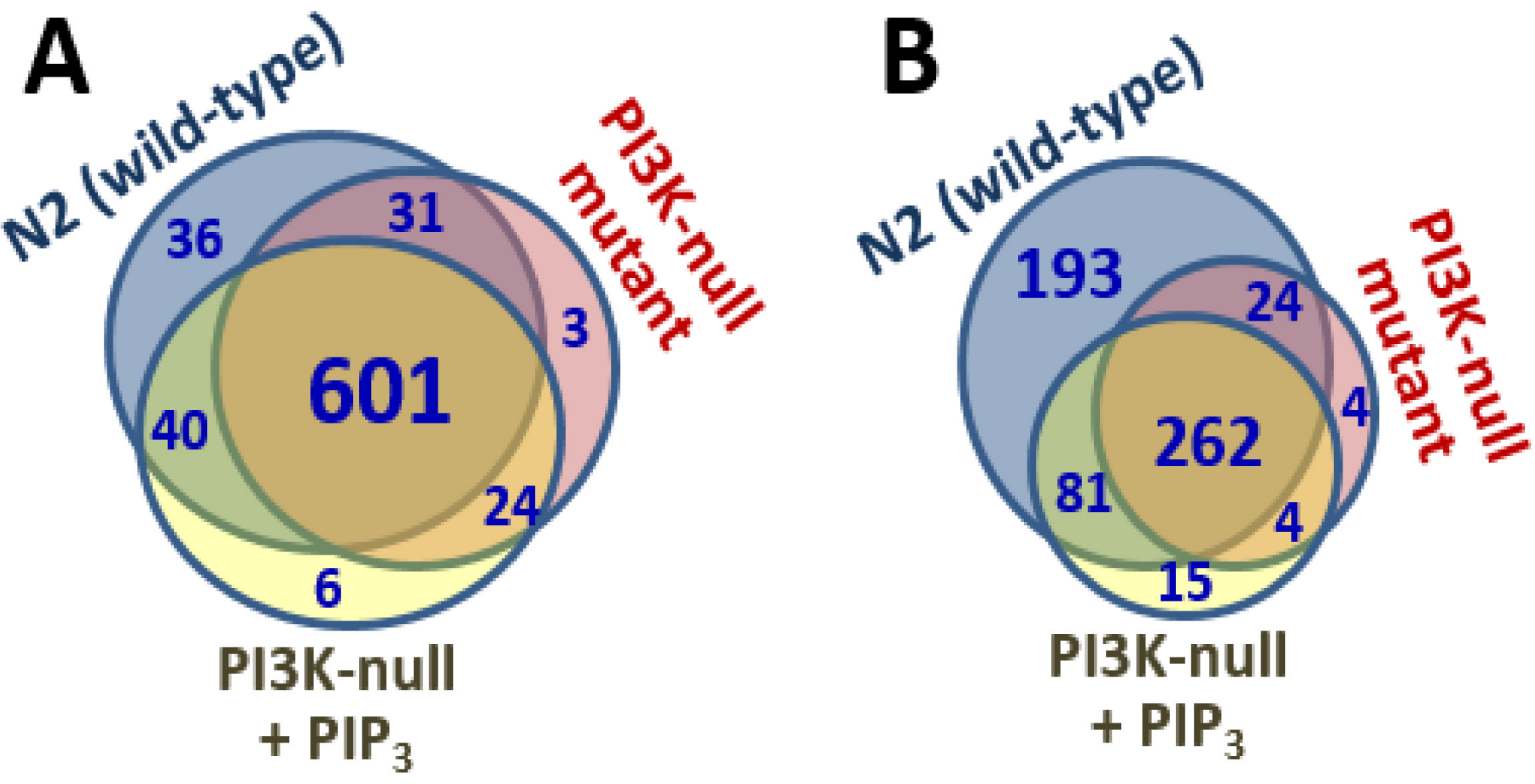
Venn diagrams Indicate numbers of proteins positively identified in each membrane fraction (**A**), or in the subset of those proteins that bound PIP_3_>>PIP_2_ (**B**)**.**

Gels like those of Figure 2 were sliced and analyzed by high-resolution proteomics to identify the proteins in each lane. Complete spectral counts are listed in Supplementary Data, Table S1, and summarized as Venn diagrams in Figure 3A. Of the 708 membrane proteins identified from N2, 632 (89%) were also seen in *age-1(mg44)* F2 adults lacking active PI3K_I_ and having no detectable PIP_3_, so only 11% of these proteins may be membrane-associated via PIP_3_ tethering. Feeding PIP_3_ to PI3K-null worms restored 40 proteins that were identified in N2 (5.6%). Considering just those membrane proteins that bind PIP_3_ far more than PIP_2_ based on relative spectral counts (Figure 3B), 560 proteins from N2 adults met these criteria but just 286 of those (51%) were also identified in very long-lived *age-1(mg44)* F2 adults. Feeding PIP_3_ restored 81 proteins found in N2 (15%), or 30% of the 274 N2-specific proteins that might have been rescued.

### Proteins that preferentially bind PIP_3_

In Table 1, results are compiled from 3 typical experiments (of 5 that were run). The table lists many proteins that consistently preferred PIP_3_ over PIP_2_ binding (Experiments 1 and 2) and/or were greatly depleted in *age-1(mg44)* worms lacking active PI3K_I_ and hence PIP_3_ (Experiment 3). Noteworthy differences are highlighted in yellow. Examples include AKT-1, a Ser/Thr kinase known to contain a PIP_3_-specific PH domain [4], which strongly preferred binding to PIP3 over PIP2 but was not effectively recovered from membranes of well-fed worms; muscle M-line assembly protein (UNC-89) and both nematode 14-3-3 proteins (PAR-5 and FTT-2), all reported to contain PH domains of uncertain specificity [4]; disorganized muscle protein 1; a variety of vitellogenins, protein precursors of LDL proteins also related to ApoB-100; chaperonins HSP60, HSP70, HSP90, and 4 subunits of the T complex, important in protein folding and refolding; protein disulfide isomerase, involved in refolding oxidized proteins; V-type proton ATPase; 3 fatty-acid binding proteins; 14 ribosomal proteins; and 5 α (noncatalytic) subunits of the 26S proteasome, most of which are in the 20S core assembly.

**Table 1:** PIP_3_-binding proteins identified in at least two experiments, based on affinity-binding capture ratios (Experiments 1 and 2) or genetic evidence (Experiment 3B)

Proteins	Genes	MW (kDa)	Expt. 1 Bound to		Expt. 2 Memb. Prot. Bound to N2		Expt. 3A Membranes			Expt. 3B, PIP_3_-Binding, from 3A			Description
			PIP_3_	PIP_2_	PIP_3_	PIP_2_	N2	*mg44*	*mg*+P	N2	*mg44*	*mg*+P	
**AKT-1, Ser/Thr protein kinase**	akt-1*	**62**	**14**	**0**	**11**	**0**	**0**	**0**	**0**	**2**	**1**	**2**	Pleckstrin Homol. Domain for PIP_3_
**Muscle M-line assembly protein**	***unc-89***	**894**	**5**	**2**	**1**	**0**	**36**	**20**	**27**	**3**	**0**	**0**	Pleckstrin Homol. Domain, PIP_2/3_
**14-3-3 proteins**	par-5**, ftt-2**	**28**	**8**	**0**	**35**	**16**	**87**	**72**	**89**	**2**	**0**	**5**	Likely PHDs binding PIP_2_ or PIP_3_
**Disorganized muscle protein 1**	***dim-1***	**72**	**3**	**1**	**2**	**2**	**17**	**13**	**14**	**6**	**0**	**0**	Associates with a PHD protein
**Vitellogenins 2, 3, 5, 6**	vit-2*,-3*,-5*,-6*	**186+**	**76**	**8**	**661**	**398**	**4054**	**4512**	**6333**	**233**	**69**	**159**	LDLs related to ApoB100
**HSP chaperones: HSP70C, HSP70D, HSP60; DAF21/HSP90**	***hsp-3***^+^, ***-4***^+^, ***-60***^+^***; daf-21***^+^	**73,72, 60,80**	**26**	**0**	**41**	**18**	**248**	**142**	**183**	**9**	**0**	**1**	Chaperonins req'd for misfolded proteins; HSP-60 is mitochondrial
**HSP70A**	***hsp-1***	**70**	**18**	**12**	**38**	**27**	**69**	**51**	**58**	**5**	**0**	**1**	
**V-type proton ATPase s.u.’s**^+^	***vha-12***^+^, ***-13***^+^, ***-15***	**55-66**	**19**	**0**	**26**	**13**	**124**	**90**	**97**	**11**	**2**	**4**	Vacuole H^+^-translocating ATPase
**Fatty-acid binding proteins**	far-1, −2; lbp-6*	**16-20**	**5**	**0**	**3**	**3**	**30**	**22**	**47**	**0**	**0**	**0**	Lipid transporters; *lbp-6* KD is LL
**Tubulin alpha-2, beta-2 chains**	***tba-2***^+^, ***tbb-2***^+^	**50**	**15**	**3**	**38**	**35**	**104**	**88**	**95**	**1**	**0**	**0**	Structural protein
**Protein disulfide isomerase 2**	***pdi-2***^+^	**55**	**4**	**0**	**5**	**1**	**86**	**73**	**75**	**3**	**0**	**0**	Role in ER folding oxidized-prots
**rRNA 2′-O-methyltransferase**	***fib-1***	**36**	**3**	**0**	**3**	**3**	**35**	**6**	**13**	**0**	**0**	**0**	Fibrillarin, part of U3 SnoRNP
**Lamin-1**	lmn-1**	**64**	**2**	**0**	**4**	**0**	**6**	**1**	**5**	**0**	**0**	**0**	Nuclear envelope structural prot
**T-complex protein 1 s.u. ϵ**	***cct-5***^+^	**59**	**1**	**0**	**15**	**4**	**20**	**1**	**6**	**0**	**0**	**0**	Chaperonin complex
**Alpha Enolase**	***enol-1***^+,^*^,^**	**47**	**1**	**0**	**9**	**3**	**50**	**29**	**43**	**0**	**0**	**0**	RNAi alters LS, reduces aggreg'n
**Adenosylhomocysteinase**	***ahcy-1***^+^	**48**	**11**	**1**	**23**	**15**	**72**	**24**	**48**	**2**	**0**	**0**	Interacts w. CCT, UBA-1, UBQ-2
**Pyruvate carboxylase 1**	pyc-1**	**129**	**35**	**10**	**32**	**28**	**46**	**39**	**57**	**39**	**37**	**43**	Reg. enz. for gluc & lipid metab.
**Methylcrotonoyl-CoA carbox.β**	**F02A9.4**	**67**	**54**	**25**	**59**	**52**	**12**	**7**	**11**	**32**	**17**	**22**	Function predicted, not proven
**60S ribosomal proteins**	***rpl-4, 5, 7–10, 20, 36***	**12-39**	**51**	**1**	**79**	**63**	**624**	**344**	**454**	**75**	**9**	**12**	Other 60S proteins not PIP_3_-spec.
**40S ribosomal proteins**	***rps-7, 12, 13, 19,23,25***	**13-22**	**16**	**1**	**0**	**0**	**213**	**107**	**149**	**42**	**5**	**7**	Other 40S proteins not PIP_3_-spec.
**Cullin-associated, NEDD8-dissociated protein 1**	cand-1*	**70**	**1**	**0**	**0**	**0**	**6**	**0**	**3**	**14**	**10**	**0**	Assembles SCF (SKP1-CUL1-F-box) /E3-ubiquitin ligase complexes
**Rad-50**	***rad-50***	**150**	**1**	**0**	**1**	**0**	**5**	**3**	**3**	**15**	**6**	**13**	HR-directed DNA DS-break repair
**Translationally-controlled tumor protein homolog**	***tct-1***	**21**	**1**	**0**	**1**	**0**	**15**	**1**	**2**	**28**	**20**	**14**	ER protein needed in developm't, growth, locomotion, reproduction
**Dynein heavy chain, cytoplasm**	***dhc-1***	**522**	**11**	**6**	**0**	**1**	**34**	**3**	**1**	**188**	**77**	**117**	Places microtubule organizing ctr
**Peroxiredoxin**	***prdx-3***	**25**	**1**	**0**	**1**	**1**	**5**	**0**	**4**	**15**	**17**	**26**	Oxidative-stress response
**Acetyl-coA acetyltransferase, mit.**	***kat-1***	**42**	**0**	**0**	**0**	**0**	**14**	**1**	**0**	**16**	**9**	**13**	Fatty-acid β-oxidation, via Sir2
**Proteasome α subunits**	***pas-1,-3,-5,-6,-7***	**28**	**1**	**0**	**10**	**2**	**17**	**11**	**17**	**7**	**5**	**6**	Proteasome structural/regul. SU's
**Fatty acid desaturases**	fat-1,-2*,-4*,-6,-7	**39-52**	**0**	**0**	**0**	**0**	**63**	**1**	**3**	**0**	**0**	**0**	These proteins (8 lipid biosynthesis enzymes, 2 MDR proteins, and 1 intermediate filament protein) are found ONLY in membrane preps & have very high N2/*mg44* ratios
**Fatty acid elongases**	***elo-3, −4, −5***	**32-38**	**0**	**0**	**0**	**0**	**18**	**1**	**0**	**0**	**0**	**0**	
**Multidrug resistance proteins**	***pgp-1, pgp-3***	**140+**	**0**	**0**	**0**	**0**	**25**	**1**	**5**	**0**	**0**	**0**	
**Intermediate filament protein**	***Ifb-1***	**67**	**0**	**0**	**3**	**4**	**18**	**0**	**7**	**0**	**0**	**0**	

Related proteins that behaved similarly have been grouped together as indicated. **Expt. 1**: Worm (N2) proteins, recovered after affinity binding to PIP_2_- or PIP_3_-coated beads, were electrophoresed on polyacrylamide/SDS gels, and identified from trypsin-digested gel slices by LC-MS/MS proteomics. **Expt. 2**: Membrane proteins were isolated from wild-type worms (**N2**), and associated proteins were recovered using a detergent that dissociates protein complexes (unlike Expts. 1 and 3). **Expt. 3A**: Membrane proteins were isolated from wild-type worms (**N2**), a PI3K-null mutant (***mg44***) or *mg44* adults fed PIP_3_. **Expt. 3B**: Proteins from **3A** were bound to PIP_3_-beads, eluted, resolved by electrophoresis, and identified from trypsin-digested gel slices by LC-MS/MS proteomics. Spectral counts are shown, indicating the number of significant peptide identifications per protein, a crude measure of *relative protein abundance*. Deep yellow highlighting indicates ratios of ≥5; lighter yellow indicates suggestive differences. *RNAi knockdown extends lifespan; **RNAi reduces lifespan; †RNAi alters protein aggregation.

Gene ontology (GO) and pathway (KEGG) terms enriched among the proteins that preferentially bind PIP_3_ (Table 2) include Translation (enriched 7.4-fold, *P* < 10^−64^), Stress response (5.9-fold, *P* < 10^−5^), Mitochondria/respiration (5.6-fold, *P* < 10^−5^), Determination of adult lifespan and Aging (3.3-fold, *P* < 10^−10^), Lipid transport (2.9-fold, *P* < 0.003), Proteasome core complex (2.8-fold, *P* < 10^−10^), and Unfolded protein response (2.8-fold, *P* < 10^−5^).

**Table 2:** GO/Pathway analysis

GO terms enriched for PIP3-specific binding proteins (total)			
Term	Count	Fold Δ	FDR
Translation	107	7.4	5E–65
Protein biosynthesis	40	2.4	2E–43
Positive regulation of growth	109	2.0	7E–25
Adult life-span determination / Aging	46	3.3	1E–10
Proteasome core complex	18	2.8	1E–10
Proteasome component region PCI	6	2.2	1E–9
Stress response	6	5.9	4E–6
Unfolded protein response	3	2.8	6E–6
Mitochondria / Cellular respiration	14	5.6	1E–5
Lipid transport	4	2.9	3E–3

Functional-annotation enrichment (https:/david.ncifcrf.gov/) was analyzed for proteins recovered from PIP3-affinity beads and identified by LC-MS/MS. Count: total proteins per category. Fold Δ: term enrichment factor. FDR: False Discovery Rate (<0.05 is considered significant).

### *In silico* modeling predicts preferential interaction with PIP_3_ over PIP_2_, for most proteins with higher observed affinity for PIP_3_

Molecular docking programs were used to estimate affinities for PIP_3_ *vs*. PIP_2_ of proteins empirically observed to prefer PIP_3_. The docking predictions are not expected to support all of the observed PIP_3_-specific candidate proteins, since retention on PIP_3_-coated beads could reflect either direct or indirect binding (e.g., via a complex). We began by retrieving crystallographic or NMR-based structures from the PDB database, for structure-defined orthologs of candidate proteins that had shown preferential affinity for PIP_3_ in multiple experiments. The corresponding *C. elegans* protein structures were then derived using molecular modeling with I-TASSER or MODELLER 9.13 (see Methods). Similarly, the NMR-based structures of PIP_3_ and PIP_2_ were refined in MODELLER, chiefly by truncating the fatty-acid chains to limit their contributions to interactions.

**Figure 4:**
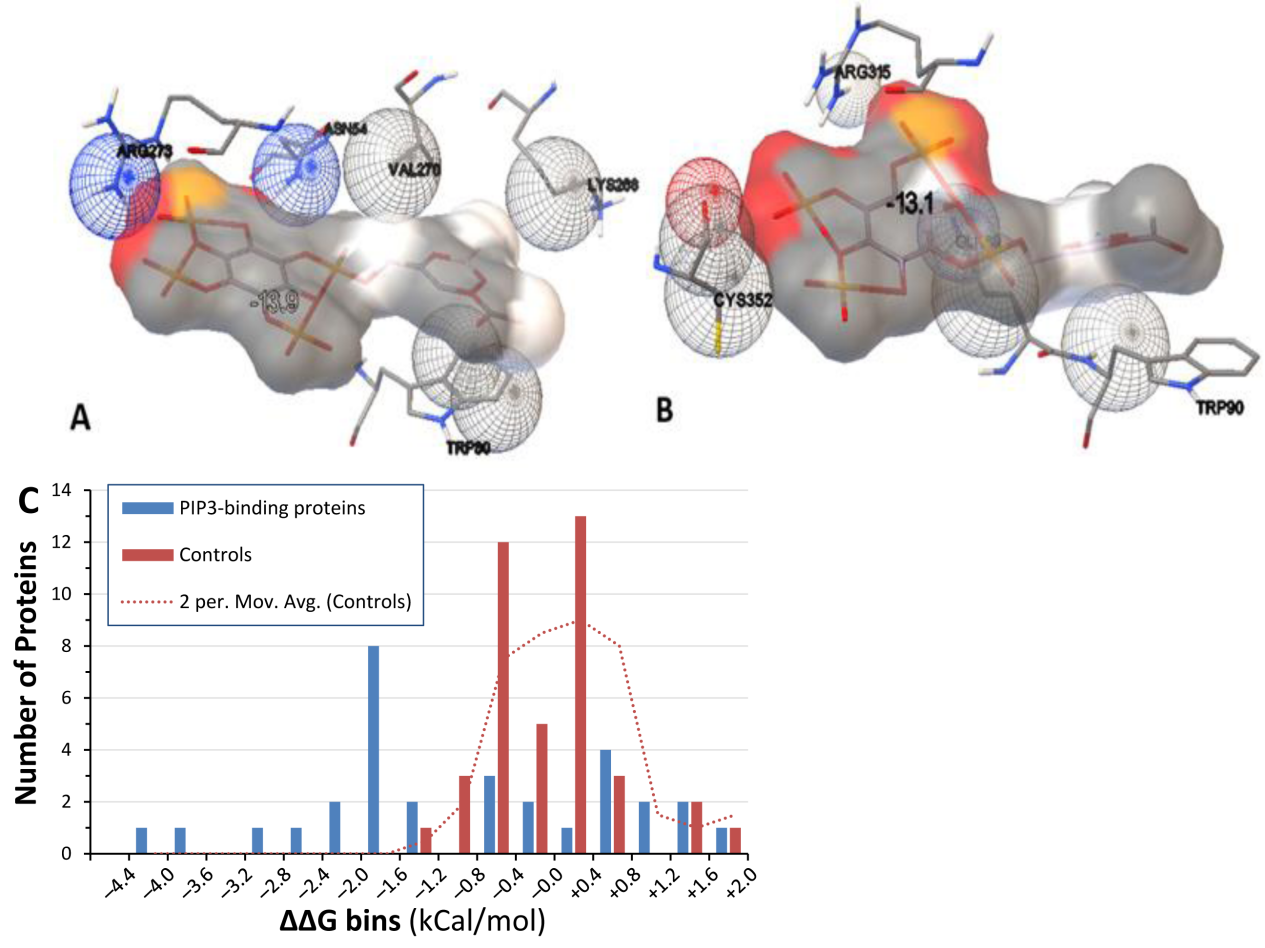
Docking models of PIP_**3**_ binding to proteins **A.** & **B.** Hybrid models of PIP_3_ (grey) and contact residues of human AKT (**A**) with ΔG_binding_ of −13.9 kCal/mol, and nematode AKT-1 (**B**) with ΔG_binding_ of −13.1 kCal/mol. **C**. “bin plot” of ΔΔG values for PIP_3_-specific binding candidates (blue bars) and randomly chosen control proteins (red bars). Intervals are rightward inclusive (e.g. proteins are shown between −1.6 and −1.2 if ΔΔG lies between −1.59 and −1.2). Dotted line: smoothed control distribution, taken as a moving average of adjacent count pairs.

Docking of each protein structure was simulated with PIP_2_ and PIP_3_ separately by energy minimization, using AutoDock-Vina to calculate ΔG_binding_ (the change in Gibbs free energy on binding) for each docking interaction. Table 3 shows ΔG_binding_ for protein binding to PIP_3_ or PIP_2_ and the difference between them. That energy difference, ΔΔG = ΔG(PIP_3_)–ΔG(PIP_2_), indicates the binding preference for either phosphoinositide. Docking models that emphasize contact points (Figure 4A & 4B) illustrate the precise fit of PIP_3_ in PH domains of both human and nematode AKT proteins. Predicted ΔΔG values for 15 of 31 candidate proteins (48%) surpassed all 40 randomly chosen control proteins (Figure 4C; rank-order *P* < 3 × 10^−4^), and 16 of those (bold lines, Table 3) would be considered significant at an empirical threshold of *P* < 0.05. For all 16, ΔG(PIP_3_) was < −7.5, indicating relatively stable interactions.

**Table 3:** *In silico* interaction energies of PIP_3_-binding proteins with PIP_3_ vs. PIP_2_

Protein [Role]	ΔG_binding_ for PIP_3_ (kCal/mol)	ΔG_binding_ for PIP_2_ (kCal/mol)	ΔΔG
CYC-2.1 [one of 2 *C. elegans* cytochrome c proteins]	−10.6	−6.5	−4.1
RPL-21 [ribosome large s.u. protein 21, SH3 domain]	−13.6	−9.8	−3.8
DKC-1 [ortho. of human dyskerin, H/ACA RNP s.u.]	−10.4	−7.3	−3.1
FAT-2 (iso. A) [Δ12 FA desaturase, increases fluidity]	−9.7	−7.2	−2.5
NKB-1 [Na^+^/K^+^-transporting ATPase s.u.]	−9.2	−7.2	−2.0
CCT-1 [α subunit of T-complex chaperonin]	−9.0	−7.1	−2.0
AKT-1c [insulin-like signaling kinase]	−10.6	−8.6	−1.9
RAD-50a (C-term.) [part of homol. recomb. complex]	−9.1	−7.3	−1.8
IFE-1 (iso. A) [mRNA cap-binding protein eIF4E]	−10.1	−8.3	−1.7
EIF-1 [ortho. of euk. translation initiation factor EIF1]	−8.7	−7.1	−1.7
PAS-1 [α subunit 1 of 26S proteasome]	−11.7	−10.0	−1.7
TCT-1 [orthol. to human TPT1, tumor protein 1]	−9.4	−7.8	−1.6
SOD-2 [Fe^++^/Mn^++^ superoxide dismutase]	−7.9	−6.3	−1.6
MSP-78 [major sperm protein 78]	−8.8	−7.3	−1.6
HSP-6 [DnaK/Hsp70 family chaperone]	−7.5	−6.2	−1.4
KAT-1 [ketoacyl-coA thiolase, part of FA β-oxidation]	−8.9	−7.7	−1.2
HIPR-1 [ortho. to Huntingtin-interacting protein 1-r]	−6.3	−5.7	−0.6
CAND-1 [cullin-associated NEDD8-dissoc. protein 1]	−5.7	−5.1	−0.6
FAR-1 (iso. A) [fatty acid/retinol binding protein 1]	−10.9	−10.5	−0.4
MSP-77 [major sperm protein 77]	−8.7	−8.4	−0.3
SOD-3 [Fe^++^/Mn^++^ superoxide dismutase, orth. SOD1]	−6.8	−6.7	−0.1
CGH-1 [DEAD-box RNA helicase]	−8.3	−8.4	0.1
SOD-1 (iso. A) [Cu^++^/Zn^++^ superoxide dismutase]	−6.2	−6.7	0.5
MSP-3 [major sperm protein 3]	−8.1	−8.7	0.6
PAS-2 [α subunit 2 of 26S proteasome]	−7.0	−7.7	0.6
LBP-1 [lipid-binding protein 1]	−8.6	−9.3	0.6
RPN-12 (iso. A) [19S proteasome, regulatory s.u. 12]	−5.5	−6.4	0.9
SODH-1 [sorbitol dehydrogenase 1]	−8.1	−9.2	1.1
IFB-1a_head [intermediate filament protein 1]	−5.9	−7.2	1.3
RPN-2 [19S proteasome, regulatory subunit. 2]	−6.7	−8.1	1.4
BCAT-1 [branched-chain aminotransferase 1]	−5.5	−7.2	1.7
**Control Proteins (top 10 proteins are shown, out of 40 taken at random from PDB)**			
1a3q	−9.8	−8.6	−1.2
1a3b 5^th^ percentile ΔΔG threshold	−7.4	−6.3	−1.1
1a10	−7.7	−6.7	−1.0
1agf 10^th^ percentile ΔΔG threshold	−8.2	−7.3	−0.9
1a00	−7.8	−7.1	−0.7
1a0z	−7.4	−6.7	−0.7
1ah1	−5.9	−5.3	−0.6
1a17	−6.0	−5.4	−0.6
1axa	−7.6	−7.1	−0.5
1a1m	−7.3	−6.8	−0.5

For each protein structure listed (obtained from PDB or derived as described), docking was simulated with PIP_2_ or PIP_3_ by energy minimization using AutoDock-Vina. ΔG_binding_ was calculated for each docking, and proteins were ranked by the difference between PIP_3_ and PIP_2_ binding energies: ΔΔG = ΔG(PIP_3_) –ΔG(PIP_2_). Control protein structures were taken at random from PDB for 40 proteins, of which the top-ranked 10 (based on ΔΔG) are shown.

### Knockdown of genes encoding PIP_3_-binding proteins enhances stress resistance

**Figure 5:**
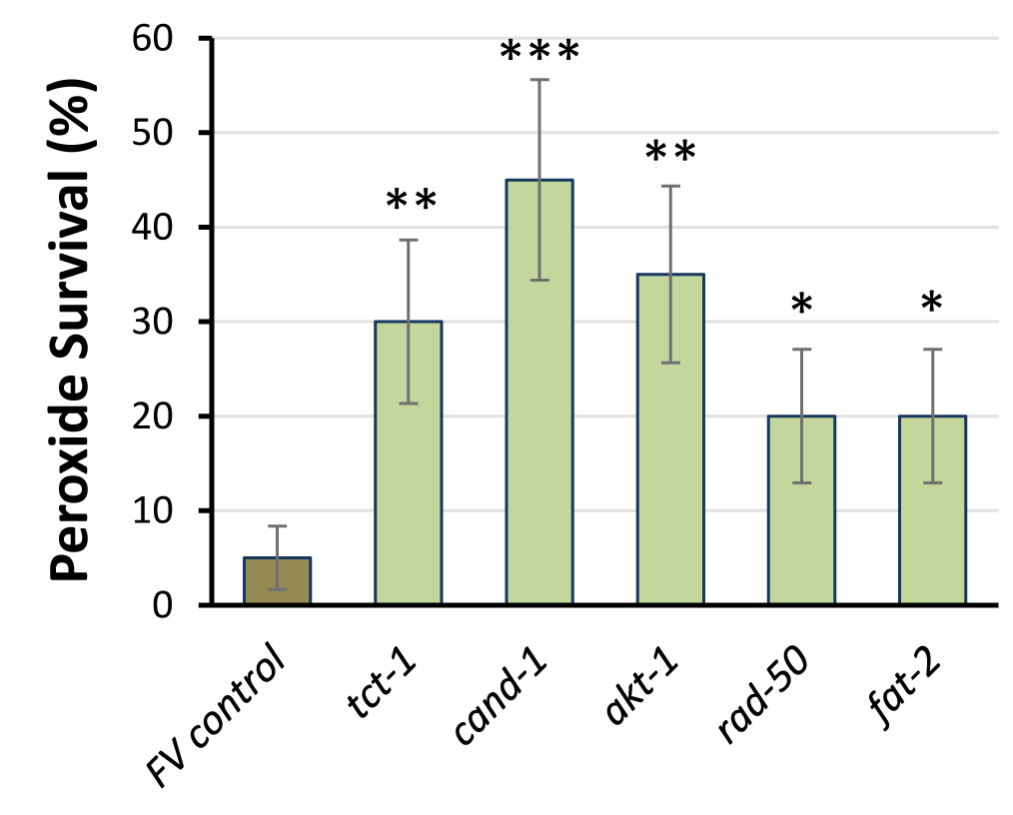
RNAi knockdown of PIP_**3**_-binding proteins improves peroxide survival Worm survival ±SE is shown after 4h in 5-mM H_2_O_2_. RNAi groups differed significantly from controls (by 1-tailed Fisher exact test): *P < 0.05; **P < 0.005; ***P < 10^−4^ (each *N* = 40). Error bars indicate the standard error of a proportion.

We selected 18 PIP_3_-binding proteins, based on function and inclusion in the Ahringer RNAi library [35], to ask whether they might contribute to the “multiple stress resistance” traits of *C. elegans age-1-*null mutants [36]. Knockdowns of 5 candidates (28%) significantly improved survival in hydrogen per-oxide (Figure 5), an oxidative stress to which *age-1(mg44)* mutants are exceptionally resistant [3]; they are TCT-1, CAND-1, AKT-1, RAD-50 and FAT-2.

### PIP_3_-binding proteins influence protein aggregation in *C. elegans* model systems

**Figure 6:**
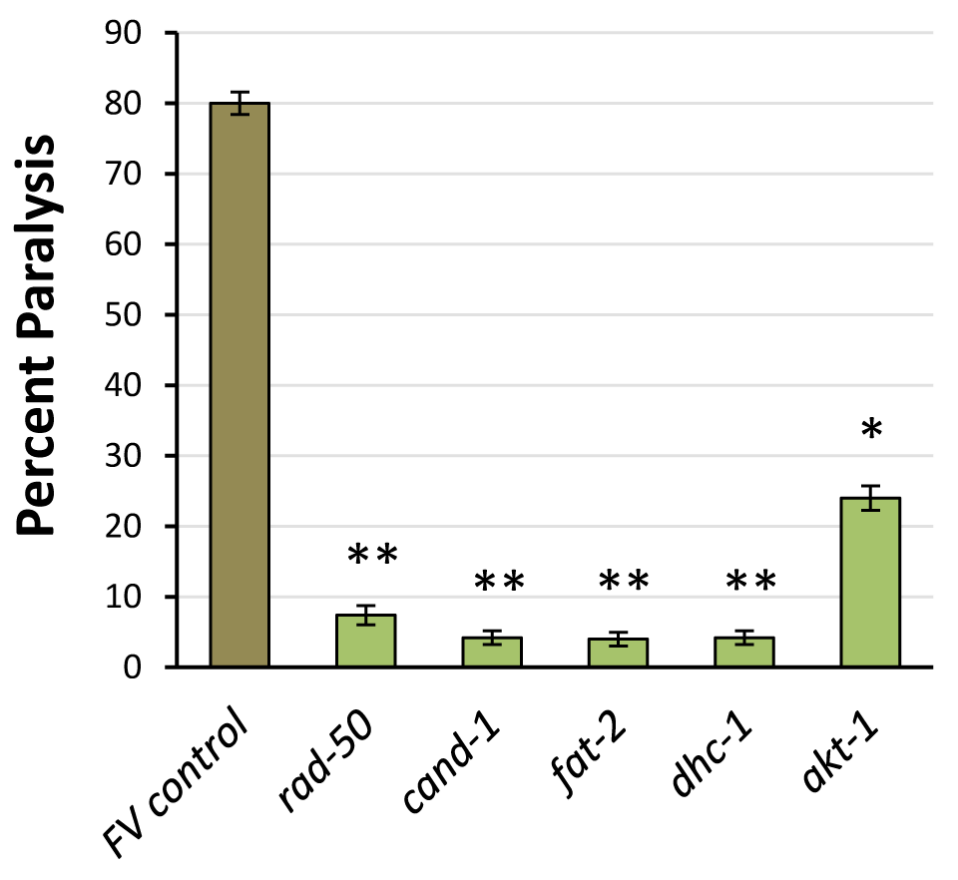
RNAi knockdown of PIP_**3**_-binding proteins reduces age-dependent paralysis from leaky muscle expression of Aβ_**1-42**_ Data show % paralyzed, ±SD, at 12 d post-hatch. RNAi groups differed significantly from controls (by 1-tailed Fisher exact test): *akt-1*, *P* < 10^−4^; all others, *P* < 10^−7^.

The same 18 PIP_3_-binding candidates were tested to determine whether they contribute to protein aggregation. Strain CL4176 can be induced to express human Aβ_1-42_ in body-wall muscle [37]; it forms β-amyloid aggregates leading to paralysis soon after induction, or progressively with age if not induced [36]. RNAi knockdown of 5 genes (28%) encoding RAD-50, AKT-1, CAND-1, FAT-2 and DHC-1, reduced age-dependent paralysis in adult worms with “leaky” Aβ_1-42_ expression [36], shown at day 12 in Figure 6. Three of these knockdowns (*rad-50, cand-1* and *fat-2*) also significantly blocked paralysis 48 h after *induction* of Aβ_1-42_ (data not shown), but we consider uninduced paralysis to be a more appropriate model of age-dependent protein aggregation.

**Figure 7:**
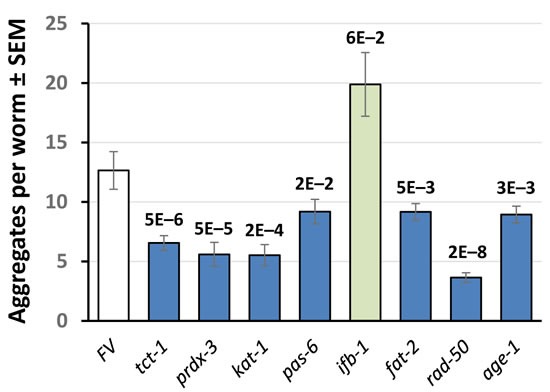
Aggregate counts for *C. elegans* **day-9 adults expressing muscle α-synuclein.** Adult worms were maintained for 9 days on bacteria expressing RNAi against the genes indicated, encoding PIP_3_-binding proteins. Error bars indicate ±SEM. Values above bars show significance of differences from Feeding Vector (FV) control, by heteroscedastic 2-tailed *t*-tests.

Muscle-specific expression of human α-synuclein (in strain NL5901), a model of Parkinson's disease, was also assessed during adult aging. Significant reductions in the number of aggregates, at least as deep as that elicited by RNAi to *age-1*, were observed at 9 and 10 days post-hatch after knockdown of genes encoding 6 (33%) of 18 PIP_3_-binding proteins tested: RAD-50, FAT-2, TCT-1, PRDX-3, KAT-1, and PAS-6 (Figure 7). KD of *ifb-1* appeared to *increase* aggregates, although without statistical significance.

### RNAi knockdowns of several PIP_3_-binding proteins confer multiple fitness benefits

**Figure 8:**
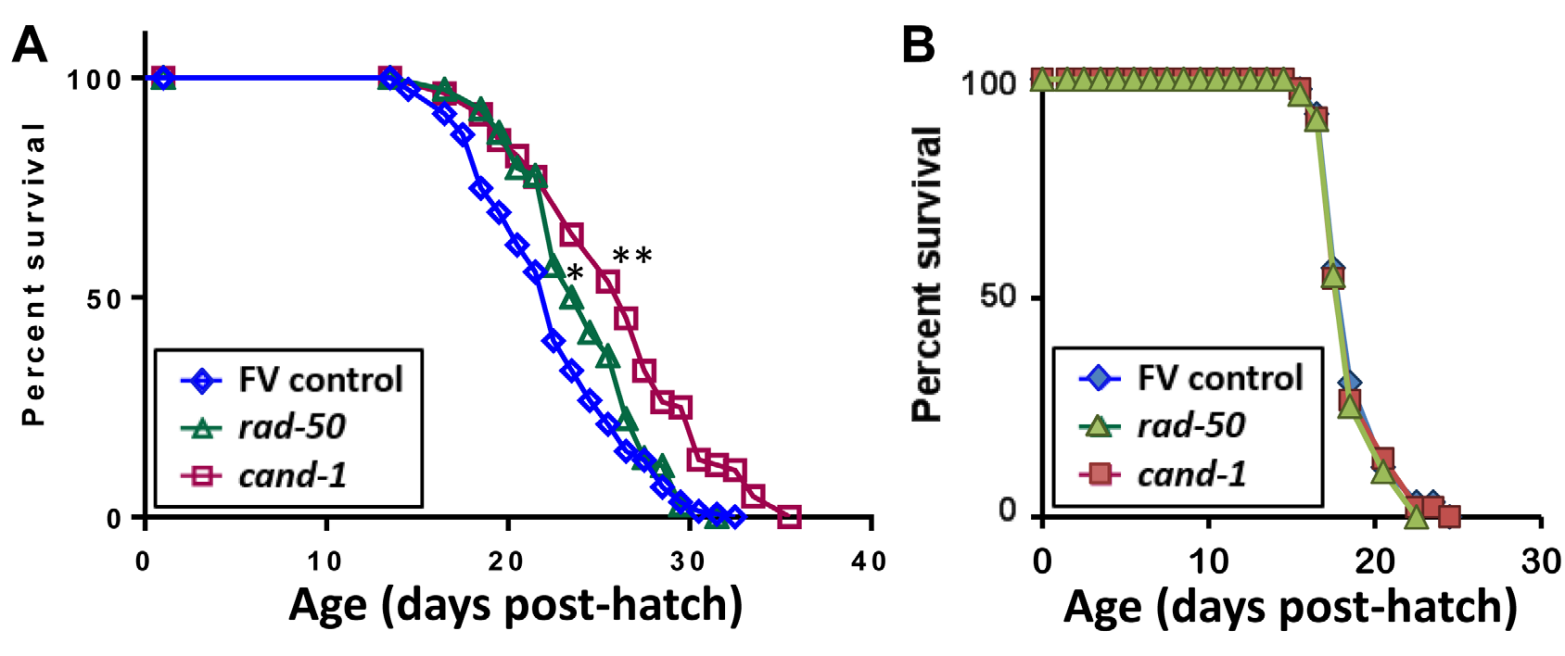
Survivals of *C. elegans* **exposed from the late-larval (L4) stage, to RNAi targeting genes that encode two PIP**_**3**_**-binding proteins.** Data were combined from 4 experiments with N2 wild-type worms (**A**) or from 2 experiments with *daf-16(m26)* mutant worms (**B**) exposed to empty feeding vector (FV) or RNAi targeting *cand-1* or *rad-50*. Total numbers of uncensored/censored deaths included were (A) control, 148/7; *cand-1* RNAi, 82/2; and *rad-50* RNAi, 114/6; and (B) control, 88/2, *cand-1* RNAi, 90/0; and *rad-50* RNAi, 87/3. *Gehan-Wilcoxon log-rank *P* < 3E-4; **Gehan-Wilcoxon log-rank *P* < 1E-4.

Previous studies in *C. elegans* had reported lifespan extension upon knockdown for two of those 5 genes, *akt-1* [38] and *fat-2* [39]. We confirmed a significant (*P* < 0.05) life extension upon *akt-1* knockdown (data not shown), and somewhat stronger effects of RNAi targeting *cand-1* (**P < 10^−4^) or *rad-50* (*P < 3×10^−4^) (Table 4, Figure 8), suggesting that these PIP_3_-binding proteins may also contribute to the unique longevity of *age-1*-null mutants that cannot form PIP_3_ [3]. Significant but less pronounced life extension was observed when RNAi was begun only at the L4 (late-larval) stage to avoid effects on development (Table 4, Expt. 4). However, no life extension was seen in a *daf-16* mutant (Figure 8B), confirming that *cand-1* and *rad-50* act via the insulin/IGF-1 signaling pathway. Of 18 PIP_3_-binding candidates tested by RNA interference, 5 improved fitness by at least two measures, survival of peroxide stress and protection in protein-aggregation models (Table 5), and at least 4 extend lifespan.

**Table 4:** Survival data for PIP_3_-binding candidate knockdowns

	Experiment 1 (N2)			Expt. 2 (N2)		Expt. 3 (N2)		Expt. 4 (N2)	
RNAi:	none	CAND-1	RAD-50	none	CAND-1	none	RAD-50	none	RAD-50
**Median**	18.5	23.5	23.5	23.5	28.5	22.5	25.5	22.5	22.5
**Ratio**	—	1.27	1.27	—	1.21	—	1.13	—	1.0
**Mean**	19.5	23.2	23.5	24.3	28.0	23.2	25.1	22.6	24.3
**Ratio**	—	1.19	1.20	—	1.15	—	1.10	—	1.08
***P* <**	—	3E–5	3E–5	—	0.0001	—	0.007	—	0.008

	Expt. 5 (N2) RNAi from L4				Experiment 6 (*daf-16*^–^)			Experiment 7 (*daf-16*^–^)		
RNAi:	none	CAND-1	RAD-50		none	CAND-1	RAD-50	none	CAND-1	RAD-50
**Median**	22.5	24.5	24.5	17.5	17.5	17.5	18.5	18.5	17.5
**Ratio**	—	1.09	1.09	—	1.00	1.00	—	1.00	0.95
**Mean**	23.5	24.5	24.6	18.9	18.6	18.4	18.5	18.6	18.5
**Ratio**	—	1.04	1.05	—	0.99	0.97	—	1.00	1.00
***P* <**	—	0.04	0.06	—	0.6	0.4	—	0.8	1.0

Median and Mean survivals are given in days post-hatch. Five independent experiments were performed in the wild-type N2-DRM strain, and two in strain SR814, created by outcrossing DR26 (*daf-16(m26)*) into N2-DRM. RNAi feeding began at hatch, or (Experiment 5) in late L4. Gehan-Wilcoxon

log-rank *P* values are shown.

## DISCUSSION

PIP_3_, the product of class-I PI3K, is normally embedded in eukaryotic cell plasma membranes where it is thought to contribute to multiple kinase-cascade signaling pathways. *C. elegans* mutants lacking the PI3K_I_ catalytic subunit exhibit extreme longevity, improved stress resistance, delayed development, and reproductive defects [3]. We infer that PIP_3_ plays critical roles in these pleiotropic physiological traits by binding and recruiting signaling proteins. Those proteins, and the pathways they participate in, must confer developmental and reproductive benefits early in life, presumably through roles in anabolic metabolism and cell proliferation. Nonetheless, they appear to have deleterious long-term consequences so that their continued activity in post-gravid adults promotes aging.

**Table 5:** Multiple benefits of PIP_3_-binding candidate knockdowns

Gene	H_2_O_2_ survival	Aβ_1_–_42_ aggreg.	α-synuclein aggregation	Longevity effect (range) or reference
		induced aging		
***rad-50***	↑5x	↓3x ↓10x	↓3.6x	↑15% (8 – 20%)
***cand-1***	↑11x	↓5x ↓20x	N.S.	↑17% (15 – 19%)
***fat-2***	↑5x	↓4x ↓20x	↓40%	↑ [40]
***akt-1***	↑9x	N.S. ↓3x	N.S.	↑9%; [45]
***tct-1***	↑7x	N.S. N.S.	↓ 2x	

N.S., not significant; grey shading, not tested here. Reference citations indicate published lifespan data.

### Proteins that bind PIP_3_ are tethered to the membrane, dependent on PIP_3_ availability

We used a combined proteomic/genetic strategy to isolate and identify PIP_3_ binding proteins in *C. elegans*. Although any affinity-capture procedure can produce false positives, we set several criteria by which to evaluate candidate proteins identified in at least 3 independent experiments.

### Preferential binding to PIP_3_ over PIP_2_

PIP_3_-specific binding was assessed by comparing the capture of any given protein based on affinity to each ligand. Many proteins showed differential binding, of which 10 (highlighted in bright yellow, Table 1/Expt. 1) met our arbitrary criteria of (*i*.) at least 5-fold higher binding to PIP_3_ than to PIP_2_ and (*ii*.) at least 5 peptide “hits”. Several caveats should be considered. The PIP_3_/PIP_2_ ratio in normal cells ranges from 0.001 to 0.02, suggesting that moderately high affinity ratios might not be sufficient to prevent binding to PIP_2_ due to its higher abundance *in vivo*. Moreover, affinity purification may not precisely mirror physiological dependence on ΔG_binding_, since binding also depends on protein abundance. Protein levels are equal for the two PI ligands in lysates, but certainly differ among cell types *in vivo*, which might also vary in their PIP_3_ levels. Binding propensities *in vivo* can also be altered by additional factors such as competing ligands, other interacting proteins in a complex, and other hydrophobic or electrostatic features in the immediate vicinity of membrane-embedded PIP_3_.

Some proteins may show preferential affinity for PIP_3_ through indirect binding, in which the protein is part of a stable complex containing a PIP_3_-specific binding protein. Our data suggest that this may be quite common. We note that Experiments 1 and 2 in Table 1 differed in only one respect: protein complexes were disrupted in the second experiment, leading to loss of many “PIP_3_-specific” proteins that had ceased to be significantly differential. The value of candidate PIP_3_-binding proteins as potential pharmacologic targets, however, does not depend on whether membrane-tethering is direct or indirect.

### Presence on cell membranes, dependent on biological availability of PIP_3_

We took advantage here of our previous studies demonstrating that PIP_3_ is essentially absent (below detectable limits, i.e. < 1% of normal levels) in second-generation homozygous *age-1* mutants lacking active PI3K_I_ or detectable PIP_3_ [2]. Thus we expected that genuine PIP_3_-binding proteins will be recovered from membranes of wild-type worms but not from extremely long-lived *age-1(mg44)* mutant worms. This expectation was met convincingly (>2-fold reduction and *P* < 0.05) by a subset of candidate proteins (Table 1, Expt. 3A): lamin-1, 2 chaperones (HSP60, HSP90), rRNA 2′-O-methyltransferase, T-complex subunit CCT-5, adenosylhomocysteinase, 11 ribosomal proteins, CAND-1, TCT-1, 5 fatty-acid desaturases and 2 elongases, 2 multidrug resistance proteins and an intermediate filament protein.

Many proteins are integral to membranes, and if these are in vast excess they might mask proteins that are recruited to membranes via tethering to PIP_3_. Thus it is reassuring that most PIP_3_-binding proteins that were affinity-isolated from the membrane fraction, although depleted in overall abundance by the two-step isolation, were further reduced in *age-1(mg44)* adult worms relative to N2 controls (Table 1, Experiment 3B). Apparent exceptions include pyruvate carboxylase 1, CAND-1, TCT-1, peroxiredoxin and proteasome α subunits.

### Restoration of membrane tethering by exogenous PIP_3_

In principle, it should be possible to reverse all “*age-1*” traits in worms genetically deficient in PI3K_I_ and hence in PIP_3_, by supplying exogenous PIP_3_, provided that it is taken up, is not degraded, and reaches all tissues. Of the proteins listed under (2.) above, fatty-acid desaturases and elongases showed essentially no reversal (≤3%) whereas all other proteins were partially reversed (17-80%) by PIP_3_ feeding.

This wide variation in efficacy of PIP_3_ supplementation appears paradoxical: If PIP_3_ is able to enter cells to reverse any of the *age-1* phenotypes, should it not reverse all of them? Variable extents of rescue could result from our use of very short-acyl-chain PIP_3_, which may be insufficient to tether large proteins or complexes to the plasma membrane. A plausible alternative explanation is tissue heterogeneity of PIP_3_ uptake, since PIP_3_ added to the medium or the bacterial lawn would reach intestinal cells first, and unless it saturated the membranes of those cells it might not distribute any further. Our data appear inconsistent with this scenario, however, since the proteins least effectively “reverted” (restored to membrane fractions in *age-1(mg44)* F2 worms) by PIP_3_ feeding are fatty-acid desaturases and elongases, expected to be concentrated in the intestine since it serves as the lipid-storage organ of nematodes [40, 41].

### Molecular modeling can predict proteins that preferentially bind to PIP_3_

Park *et al*. [5] used “machine learning” to define the amino-acid residues and positional constraints within PH domains that distinguished between proteins demonstrated previously (or, for the test group, demonstrated in that paper) to have binding specificity for particular PI moieties including PIP_3_. Apart from that study, no systematic attempt has been made to identify all proteins with PIP_3_-specific binding. Over the last few years, molecular modeling has proven itself capable of screening protein-drug and protein-protein interactions with ever-increasing reliability. Nevertheless, we were uncertain whether it would prove equal to the challenge posed by discriminant PIP_3_-binding.

Just over half of the 31 tested proteins that had been identified as strong PIP_3_-specific candidates substantially exceeded the minimal energetic requirements for avid and selective binding of PIP_3_, with ΔΔG values outside the range observed in randomly-chosen control proteins (Table 4; Figure 4C). We note that PIP_3_ would be considered a reasonable candidate ligand for all 31 proteins, given that their ΔG levels ranged from −5.5 to −13.6; protein ligands predicted previously by AutoDock agreed well with confirmed binders provided that ΔG_binding_ was < −4 [42]. Our results strongly support the premise that molecular modeling can predict substrate specificity for PtdInsP-binding proteins. The 15 proteins with unexceptional ΔΔG values may not selectively bind PIP_3_ themselves, but instead participate in larger protein complexes that include a PIP_3_-specific component. Such indirect tethering of complexes may be quite common, but the candidate proteins nevertheless remain drug targets of interest if they are components of complexes that require PIP_3_ or membrane localization to function.

### PIP_3_-binding proteins are enriched for roles in translation, longevity and proteostasis

Interesting proteins that met multiple criteria for PIP_3_-specific binding include AKT signaling kinases (AKT-1, AKT-2); heat-shock chaperones (HSP60, HSP70s, and HSP90); T-complex protein CCT-1; 14-3-3 proteins (PAR-5, FTT-2); muscle M-line protein UNC-89; branched-chain amino-acyl transferase BCAT-1; ubiquitinylation/NEDD8 regulator CAND-1; RNA helicase CGH-1; proteasome subunits (PAS-1, PAS-2, RPN-7); 8 fatty-acid desaturases and elongases (FAT and ELO proteins), and 14 ribosomal proteins (8 RPL and 6 RPS).

Among these proteins are several previously reported to possess or associate with PIP_3_-binding domains (AKT [43]; 14-3-3 proteins [44]; DIM-1; UNC-89 [www.wormbase.org]), and a remarkably large number that were previously implicated in longevity and age-related diseases: AKTs [45]; HSPs [46]; 14-3-3 proteins [44]; fatty acid desaturases FAT-2 [39] and FAT-4 [40]; lipid-binding protein LBP-6 [47]; pyruvate carboxylase, PYC-1 [39]; α-enolase/ ENOL-1 [48]; and lamin-1 [49]. In addition, many have roles in proteostasis failure leading to protein aggregation, a process that is critical to lifespan regulation [36,50]: HSPs [46]; T-complex proteins [51]; V-type proton ATPase [51]; actin and tubulin chains [52]; disulfide isomerase [53]; DHC-1 [54]; adenosylhomocysteinase [51]; and proteasomes [36]. Dynein heavy chain 1 (DHC-1) is associated with neurodegenerative diseases and protein aggregation [54], and ketoacyl thiolase (KAT-1) is a conserved mitochondrial fatty-acid β-oxidase that delays aging, independent of known longevity pathways [www.wormbase.org].

The frequent recovery of ribosomal proteins, specific to PIP_3_ affinity and to worms with PI3K_I_ activity, suggests that PIP_3_ may be involved in tethering ribosomes to rough endo-plasmic reticulum. This conjecture is currently supported only by indirect evidence [55], but would account for the 7.4-fold GO enrichment for “translation” (Table 2).

### PIP_3_-binding proteins contribute to diverse pathways mediating extreme *age-1* traits

Although it was not a criterion for PIP_3_-specific binding, a goal of the current study was to assess whether any of the proteins downstream of PI3K_I_, if depleted by RNA interference, could confer some part of the beneficial survival traits displayed so strikingly by very-long-lived *age-1*-null mutant worms [3]. In fact, as shown in Figure 5, RNAi directed against genes *rad-50, cand-1, cct-1, fat-2*, and *akt-1*, encoding candidate PIP_3_-binding proteins, extended the length of time that adult worms could survive a lethal oxidative stress (5-mM H_2_O_2_), emulating the greatly enhanced peroxide-resistance of *age-1(mg44)* F2 adults [3]. Moreover, RNAi targeting r*ad-50, cand-1, fat-2*, and *dhc-1* rescued 92-100% of the paralysis that otherwise progressively afflicted worms expressing Aβ_1-42_ in body-wall muscle (Figure 6). RNAi directed against *akt-1*, although also significant, was less effective (69% rescue) ― perhaps due to functional redundancy between AKT-1 and AKT-2 proteins. Two of these RNAi treatments were previously reported to increase longevity, and three (targeting *akt-1, cand-1* and *rad-50*) produced moderate increases in lifespan (9-20%) in our hands.

In view of the importance of PI3K_I_ as a driver of cell proliferation, inhibitors have been actively sought as potential chemotherapeutic agents for cancer. In pursuit of novel anti-cancer drugs, and equally in seeking drugs to prevent or ameliorate Alzheimer's and other age-dependent diseases, potential therapeutic benefits of PI3K_I_ inhibitors have been over-shadowed by the virtual certainty that they would also be detrimental to stem cell niches. This concern has motivated our search for PIP_3_-binding proteins as alternative targets downstream of PI3K_I_. Among these, AKT is believed to drive most of the proliferative effects of PI3K [45], whereas RAD50, CAND1, FAT2 and TCT1 constitute novel targets that may preserve survival benefits of PI3K_I_ disruption, uncoupled from blockage of cell proliferation.

## MATERIALS AND METHODS

### *C. elegans* strains and culture

Wild-type Bristol N2 [DRM stock], DR26 [*daf-16(m26)*], AM141 (*unc-54p*/Q40:: YFP), CL4176 (*smg-1*^ts^ [*myo-3*/Aβ_1-42_/long 3′-untranslated region (UTR)]), and NL5901 (*unc-54p*/α-synuclein::YFP), were obtained from the Caenorhabditis Genetics Center (CGC). Strain SR808 was created by outcrossing *age-1(mg44)* 6x into the N2-DRM background; strain DR26 was similarly outcrossed to create strain SR814 [3]. All strains were maintained at 20°C on 2% (w/v) agar plates containing nematode growth medium (NGM), overlaid with *E. coli* strain OP50. If induction is indicated, strain CL4176 was upshifted to 25°C at the L3-L4 transition to induce expression of the human Aβ_1-42_ transgene. To generate synchronized worms, well-fed *C. elegans* were lysed at day 3 post-hatch (adult day 1) to release unlaid eggs, which were plated on 100-mm Petri dishes containing NGM-agar seeded in a central area with *E. coli* (OP50) as described [3].

### RNA interference

Targeted genes were subjected to RNAi knock-down by feeding worms (either from the time of hatching, or from the L4 (last larval) stage to avoid developmental effects of RNAi) on HT115 bacterial sublines from the Ahringer RNAi library [56]. Briefly, synchronized eggs were recovered after alkaline hypochlorite lysis and transferred to plates seeded with HT115 (DE3) bacteria, deficient in RNAse III and containing (a.) IPTG-inducible T7 RNA polymerase, and either (b.) the L4440 plasmid with a multiple cloning site (MCS) between two inward-directed T7 RNA polymerase promotors for “feeding vector (FV) controls”, or (c.) L4440 containing an exonic segment of the targeted gene, cloned into its MCS [56].

### Isolation of membrane proteins

Synchronized day-3 *C. elegans* adults were collected after washing in S buffer, drained of excess liquid, and flash frozen in liquid nitrogen. The worm pellets were pulverized with a dry-ice-cooled mortar and pestle, and suspended in buffer with nonionic detergent (20-mM Hepes pH 7.4, 300-mM NaCl, 2-mM MgCl_2_, 1% NP40), and protease/phosphatase inhibitors (MilliporeSigma, Darmstadt, Germany) at 0°C. Worm or cell debris was removed by brief centrifugation of lysate (5 min. at 3000 rpm). Native membrane-associated proteins were isolated from lysates with ProteoExtract membrane purification kit (MilliporeSigma) following the manufacturer's protocol, and either (a.) used for PIP_3_ binding (see next section), or (b.) suspended in Laemmli buffer containing 2% SDS (w/v) and 0.3-M β-mercaptoethanol, heated 5 min at 95°C to dissolve proteins, and electro-phoresed on 4–20% polyacrylamide gels (SDS-PAGE). Gels were stained with SYPRO Ruby (ThermoFisher) to visualize total protein.

### Isolation of PIP_3_-binding membrane proteins

Isolated membrane proteins were pre-adsorbed to uncoated control beads; the unbound fraction was collected and incubated 6 h at 4°C with PIP_2_- or PIP_3_-coated agarose beads (echelon, Salt Lake City, UT). After extensive washing, bound proteins were eluted from PIP_2_- and PIP_3_-coated beads, suspended in Laemmli buffer containing 2% SDS (w/v) and 0.3-M β-mercaptoethanol, and heated 5 min at 95°C to dissolve proteins prior to separation on 4–20% polyacrylamide/SDS gels as above.

### Identification of membrane and/or PIP_3_-binding proteins

Proteins isolated from membranes, or membranes followed by PIP_3_-coated beads, were dissolved in Laemmli buffer as described above, and separated in one dimension on 1% SDS, 4–12% acrylamide gradient gels. They were then stained with SYPRO Ruby (ThermoFisher) or Coomassie Blue to visualize total protein, and 1-mm slices were excised. Proteins were digested *in situ* with trypsin, and peptides analyzed by high-resolution LC-MS/MS with a Thermo-Velos Orbitrap mass spectrometer (ThermoFisher) coupled to a nanoACQUITY liquid chromatography system (Waters, Milford MA) as previously reported [20]. Proteins were identified by MASCOT (www.matrixscience.com) matching of peptide fragmentation patterns to a database of previously observed fragment patterns [20].

### PIP_3_ feeding to “rescue” *age-1*-null worms

Very long-lived *age-1(mg44)* mutant worms are maintained as genetically mixed cultures, with a recessive visible-trait marker (*dumpy*) on a balancer chromosome carrying wild-type *age-1*. Synchronized worms that are not *dpy/dpy* (and thus not *age-1*^+/+^) are placed singly on individual nutrient-agar plates and classified based on the developmental rate of their progeny: heterozygotes (*mg44*/+) produce a majority of offspring that develop normally, reaching adulthood in 2.5 days, while all progeny of first-generation *mg44*/*mg44* homozygotes are second-generation “F2” *age-1(mg44)* homozygotes that uniformly develop quite slowly (>8 days at 20°C, from hatch to the L4/adult moult [3]). These F2 mutants were fed 30-μM phosphatidylinositol 3,4,5-triphosphate di-C4 (echelon), beginning at 3 days post-hatch (as soon as they could be distinguished with certainty from their less long-lived siblings). PIP_3_ di-C4 differs from normal PIP_3_ by having very short (4-carbon) fatty-acid chains to improve water solubility.

### Paralysis assays

CL4176 worms were synchronized as described above, and eggs were transferred to 60-mm NGM-agar plates seeded with either FV-control bacteria or RNAi-expressing bacteria to target each gene that encodes a protein of interest*.* Paralysis of worms with muscle expression of Aβ_1-42_ was assayed as described previously [36]. Briefly, worms were upshifted from 20 to 25°C at the L3-L4 transition, and triplicate groups of 50–100 worms were scored 29 h later. Alternatively, age-dependent paralysis was monitored over 10-13 days post-hatch, maintaining worms at 20°C without upshift. Paralysis was defined by movement of the head, but not the body, in response to a touch stimulus.

### Aggregation assays for Q40::YFP and α-synuclein::YFP

AM141 worms with muscle expression of an *unc-54p*/Q40::YFP transgene [27] were synchronized and fed from hatch with RNAi targeting each individual gene that encodes a PIP_3_-binding protein, or empty feeding vector (FV controls). They were assessed by imaging yellow fluorescence, 4 days post-hatch as described [36, 56]. NL5901 worms, with muscle expression of an *unc-54p*/α-synuclein::YFP fusion protein [57], were grown on RNAi (or FV) bacteria as described above and imaged at days 9 and 10 post-hatch. To record images, worms were immobilized on glass slides in S buffer containing 0.3% (w/v) sodium azide to block muscle contraction, and fluorescence images were captured on a DP71 camera mounted on a BX51 fluorescence microscope (Olympus, Tokyo) with a 10x objective. YFP-containing aggregates (fluorescent foci) were counted for >15 worms (5-10 fields) per group with dotcount (reuter.mit.edu/software/ dotcount). Statistical significance of differences in counts/worm were based on 5-10 fields per group, by 2-tailed heteroscedastic *t* tests.

### Longevity survivals

Synchronized eggs (or L4 larvae, to avoid developmental effects) were plated on control bacteria containing an empty feeding-vector plasmid, or on RNAi bacteria selected from the Ahringer library [56] transcribing double-stranded RNA of an exonic segment from a PIP_3_-binding protein. Worms were transferred to fresh plates daily for 7 days, and on alternate days thereafter, scoring worms as alive if they moved spontaneously or in response to gentle prodding [3]. Worms lost for reasons other than natural death were censored (removed from mortality calculations) from the date of first annotation onward.

### Hydrogen peroxide stress survivals

Worms were hatched and maintained on RNAi or control plates as in the preceding section. Day-1 adult worms (50 worms, 24 h after the L4/adult molt) were washed free of bacteria and incubated at 20°C in 300 μl of 5-mM H_2_O_2_ in a 24-well plate. Worms were scored at hourly intervals for survival based on movement in response to touch [36] to assess each RNAi for effect on H_2_O_2_-stress survival.

### Computer modeling of protein structures

Sequences of all proteins were compiled from WormBase and UniProt databases, and used to perform BLASTP searches to retrieve known structures from PDB (Protein Data Base). The 3-dimensional structures of candidate proteins were modeled in two ways. In the case of nematode proteins for which a reasonable template exists (i.e. ≥70% identity to a protein of known structure), template modeling was performed in MODELLER 9.13 (salilab.org/modeller) with default parameters [36]. Proteins lacking good templates were modeled in I-TASSER (zhanglab.ccmb.med.umich.edu/I-TASSER) to predict structures by *ab initio* “multi-threading” methods [58]. Ramachandran plots were generated for all structures generated in Modeller or I-TASSER using the RAMPAGE server (mordred.bioc.cam.ac.uk/~rapper/rampage.php), and amino acids in dis-allowed regions were loop-refined using MODELLER. This process was continued iteratively until the entire plots fell within permitted limits. The final minimum-energy conformers were then used for further docking analyses as described.

### Protein docking to PIP_3_ and PIP_2_

The structure of PIP_3_ (Pubchem database) was processed by truncation of fatty-acyl chains using ChemSketch, and hydrogen atoms added as required with MGL Tools and Python Molecular Viewer while monitoring torsions. The PIP_2_ structure was then generated from that of PIP_3_ by removing the 3-phosphate. AutoDock Vina 4.2 (http://vina.scripps.edu) was used with default parameters [59] to predict interactions of candidate proteins with PIP_3_ and PIP_2_, and to score the complexes for interaction energies. For control interactions, protein structures were retrieved at random from the PDB database (http://www.rcsb.org). Heteroatoms (ligand), if present in downloaded structures, were manually removed before docking, as above, in AutoDock Vina 4.2 (run with Raccoon interface on a 32-core Linux cluster). To avoid bias, entire proteins were made available for docking. Grid dimensions were defined manually for each interaction. The Gibbs free energy of binding was calculated as ΔG_binding_ = ΔG_vdW_ + ΔG_elec_ + ΔG_H-bond_ + ΔG_desolv_ + Δg_tors_, where ΔG_vdW_ = the Lennard-Jones van der Waals potential with 0.5Å smoothing; ΔG_elec_ = the Solmajer-Mehler distance-dependent dielectric potential; ΔG_H-bond_ = hydrogen-bonding potential with Goodford directionality; ΔG_solv_ = charge-dependent version of Stouten pairwise atomic solvation energy; and Δg_tors_ is a function of the number of rotatable bonds in the ligand only (see http://autodock.scripps.edu for details).

### Statistical analyses

Differences between groups were assessed for significance by the Fisher-Behrens heteroscedastic *t* test (appropriate to samples of unequal or unknown variance). Differences in relative peptide abundance, based on spectral counts relative to the total per sample, were assessed for significance by chi-squared or Fisher exact tests. Significance of longevity-survival differences was ascertained by Gehan-Wilcoxon log-rank tests. Significance of differences in peroxide-stress survival was assessed by Fisher exact tests at the earliest assay time for which control survival was < 15%.

## SUPPLEMENTARY MATERIALS TABLE


